# Risk factors for hospitalization and death due to COVID-19 among frail community-dwelling elderly people: a retrospective cohort study

**DOI:** 10.1590/1516-3180.2021.0649.R1.20122021

**Published:** 2022-08-12

**Authors:** Daniela Castelo Azevedo, Fernando César Menezes Assunção, Mônica Silva Monteiro de Castro, Estevão Alves Valle

**Affiliations:** I MD, PhD. Physician and Research and Data Science Head, Clínica Mais 60 Saúde, Belo Horizonte (MG), Brazil.; II MD. Physician, Clínica Mais 60 Saúde, Belo Horizonte (MG), Brazil; and Chief Executive Officer, LifeCode^TM^ Information System, Belo Horizonte (MG), Brazil.; III MD, PhD. Postdoctoral Research Group Member, Health Policy and Social Protection Research Group, Instituto René Rachou, (FIOCRUZ Minas), Belo Horizonte (MG), Brazil.; IV MD, PhD. Chief Medical Officer, Clínica Mais 60 Saúde, Belo Horizonte (MG), Brazil.

**Keywords:** Frail elderly, Hospitalization, COVID-19, Risk factors, Frailty, Older adults, Community-dwelling elderly, SARS-CoV-2 virus

## Abstract

**BACKGROUND:**

Advanced age, multiple chronic diseases and frailty have been correlated with worse prognosis among coronavirus disease 2019 (COVID-19) inpatients.

**OBJECTIVE:**

To investigate potential risk factors for hospitalization and death due to COVID-19 among frail community-dwelling elderly people.

**DESIGN AND SETTING:**

Retrospective cohort study of patients followed up at a geriatric outpatient clinic in Belo Horizonte, Minas Gerais, Brazil.

**METHODS:**

The associations of demographic characteristics (age and sex) and clinical characteristics (frailty, multimorbidity, number of medications with long-term use, obesity, smoking, diabetes mellitus, pulmonary diseases, cardiovascular diseases, cerebrovascular disease, and chronic kidney disease) with the risk of hospitalization and death due to COVID-19 were explored using a multivariable logistic regression model.

**RESULTS:**

5,295 patients (mean age 78.6 ± 9.4 years; 72.6% females) were included. After adjustments, the number of medications with long-term use was found to increase the odds of hospitalization due to COVID-19 (odds ratio, OR: 1.13; 95% confidence interval, CI: 1.06-1.22). Frailty, multimorbidity and diabetes mellitus also increased the odds of hospitalization (OR: 1.06, 95% CI: 1.02-1.09; OR: 1.17, 95% CI: 1.09-1.26; and OR: 2.27, 95% CI: 1.45-3.54, respectively) and the odds of death due to COVID-19 (OR: 1.07, 95% CI: 1.00-1.14; OR: 1.16, 95% CI: 1.03-1.32; and OR: 2.69, 95% CI: 1.79-6.14, respectively).

**CONCLUSIONS:**

Multimorbidity, frailty and diabetes mellitus increased the odds of hospitalization and death due to COVID-19 and the number of medications with long-term use increased the odds of hospitalization due to COVID-19 among frail community-dwelling elderly people.

## INTRODUCTION

The coronavirus disease 2019 (COVID-19) pandemic remains a major global public health problem. The World Health Organization (WHO) registered over 153 million cases and three million deaths up to the beginning of May 2021.^1^ In Brazil, there have been more than 500,000 deaths due to this disease and, recently, there has been a sharp increase in the number of cases (a “second wave”), which has put a lot of pressure on the healthcare system, with occupation rates of more than 90% in intensive care units in many Brazilian states.^2^

Advanced age and several diseases, such as diabetes mellitus, chronic kidney disease, cardiovascular disease and chronic respiratory disease have been correlated with worse outcomes, such as hospitalization, need for invasive ventilation and mortality due to COVID-19.^3–6^ Furthermore, in addition to older age and presence of chronic diseases, it is also important to consider frailty and multimorbidity, particularly among the elderly.^4^

Frailty is characterized by decreased strength, resistance and physiological response, which translates into faulty reestablishment of homeostasis after a stressing event, thus leading to a high risk of incapacity.^7^ Studies on patients older than 60 years of age have demonstrated that frailty is associated with a higher risk of death^4^ and with greater disease severity among patients hospitalized due to COVID-19.^8^ Similarly, multimorbidity, which consists of co-occurrence of multiple diseases or clinical conditions in one person,^9^ is a factor to be considered among older adults with COVID-19, since the presence of multiple chronic health problems may be related to unfavorable clinical outcomes, such as hospitalization and death.^10^

Associations and impacts of chronic diseases, frailty and multimorbidity in relation to the outcomes from severe acute respiratory syndrome coronavirus 2 **(**SARS-CoV-2) infection have been studied especially among hospitalized patients.^4,8,10,11^ For example, in one systematic review with meta-analysis that revealed that hypertension, diabetes mellitus, cardiovascular diseases and chronic respiratory diseases were morbidities that increased the risk of developing greater severity of infection by SARS-CoV-2, only studies on hospitalized patients were considered.^11^

## OBJECTIVE

The aim of this study was to investigate which demographic and clinical characteristics, including frailty and multimorbidity, were associated with higher risk of hospitalization and death due to COVID-19 among frail elderly people who were followed up at a primary healthcare facility in Belo Horizonte, state of Minas Gerais, Brazil.

## METHODS

### Design and sample

A retrospective cohort study was conducted using data from an electronic medical record system (LifeCode version 2021-03-25, EFG Inteligencia e Consultoria Ltda, Belo Horizonte, Brazil) that was specially developed for primary healthcare patients (≥ 60 years old) who were being followed up at a geriatric outpatient clinic (for patients ≥ 60 years old) delivering healthcare for patients with private insurance plans in Belo Horizonte.

At this clinic, patients are followed up monthly, either in person or remotely, by members of a multidisciplinary team (doctors, nurses, physical therapists, psychologists, nutritionists, phono-audiologists and pharmacists). The data relating to the appointments are registered in these electronic medical records that were developed for following up older patients. Information on conditions and health events relevant to this population, such as diagnosis, medications with long-term use, laboratory test results, emergency department visits, hospitalizations and death is systematically registered. The multidisciplinary team contractually undertakes filling out the electronic medical records completely and assertively while working at the clinic. The data collected is stored in the Microsoft SQL server and is made available for research when requested.

All patients with valid data who were followed up from March 15, 2020, to April 15, 2021, were included in the present study.

The present study was approved by the research ethics committee of Associação Evangélica Beneficente de Minas Gerais and by the National Research Ethics Committee (CEP 3.843.183 and CAAE 28980120; date: February 17, 2020) and forms part of a cohort study that has investigated the health of community-dwelling elderly people. All participants signed a written informed consent statement after they had been informed of the nature and details of the study.

### Assessment of COVID-19, hospitalization and death due to COVID-19

Since the first case of COVID-19 in Belo Horizonte, which was detected on March 16, 2020,^12^ patients have been advised to call the clinic if they were exhibiting any of the symptoms that have been linked to COVID-19 (fever, coughing, fatigue, loss of smell and taste, difficulty in breathing, mental confusion or chest pain).^13^ Patients are evaluated by telephone by a nurse and, if symptoms suggestive of COVID-19 are confirmed, an in-person appointment is scheduled. Whenever necessary, tests are made, and in severe cases, the patient is hospitalized.

Besides this approach, the multidisciplinary team asks about infection, emergency department visits and hospital admissions due to COVID-19, at periodic contacts with patients or with the person responsible for the patient. These events and dates are entered into the medical records. Tests performed on these occasions, as well as the hospital discharge summaries, are logged and attached to the records. Deaths among any patients followed up are informed monthly through the health insurance plan. The team assesses the families in cases of death, to record the causes and circumstances

In the present study, a diagnosis of COVID-19 registered in the medical records was considered to be an incident case of the disease. Similarly, hospitalization and death due to COVID-19 were considered from the registrations of these events in the medical records.

### Assessment of demographic and clinical characteristics

Sex, age and clinical characteristics were evaluated regarding the risks of hospitalization and death due to COVID-19. Among the clinical characteristics, frailty, multimorbidity, long-term use of medications, obesity, smoking, diabetes mellitus (DM), respiratory diseases, cardiovascular diseases, cerebrovascular disease and chronic kidney disease (CKD) were considered.^5,14^

Frailty was evaluated using the Clinical-Functional Vulnerability Index - 20 (Índice de Vulnerabilidade Clínico Funcional, IVCF-20). This is a multidimensional instrument that assesses aspects of the health of older adults through 20 questions distributed into eight sections: age, self-perception of health, everyday activities, cognition, mood, mobility, communication and multiple comorbidities.^15^ Each section has a specific score and these add up to a maximum of 40 points. The higher the resulting value is, the higher the clinical-functional vulnerability risk of the older adult also is. Total scores higher than 7 characterize older adults as frail.^15^ This instrument has been found to be a valid and reliable measurement for assessing frailty among community-dwelling patients ≥ 60 years of age.^15^

Multimorbidity was measured through the number of medical diagnoses. The diagnosis count has been commonly used to assess multimorbidity within primary healthcare.^16^ The number of medications with long-term use was evaluated as listed in the medical record. Weight and height were taken from the mobility section of IVCF-20.^15^ In this section, these measurements are recorded and the body mass index (BMI, in kg/m^2^) is calculated. Patients were considered to be obese if they had BMI ≥ 30.^17^

Smoking, DM and CKD were evaluated from their diagnoses in the medical records. Respiratory diseases were assessed through diagnoses of asthma and/or chronic obstructive lung disease. Cardiovascular disease was assessed through any of the following diagnoses: cardiomyopathy, coronary disease with or without previous acute myocardial infarction, heart failure with preserved ejection fraction and congestive heart failure. Cerebrovascular disease was assessed through a diagnosis of previous stroke.

### Statistical analysis

Descriptive analyses were conducted using frequencies and percentages (%) for categorical variables and using means and standard deviations (SD) for continuous variables.

Univariate and multivariable multinomial logistic regressions were used to investigate individual associations of demographic and clinical characteristics with the risks of hospitalization and death due to COVID-19. Variables with P-values of 0.05 or less in the univariable analysis were included in the multivariable analysis and adjusted for age, sex and frailty level (IVCF-20); frailty was corrected only for sex and age.

The results were presented as odds ratios (OR) and 95% confidence intervals (CI). All analyses were performed using STATA (version 14.1, StataCorp LP, College Station, Texas, United States).

## RESULTS

A total of 5,295 patients were included, of mean age 78.6 ± 9.4 years, and most of them were women (72.6%). The mean IVCF-20 score was 16.8 ± 6.9), the mean number of diagnosis was 5.4 ± 3.0) and the mean of number of medications with long-term use was 5.2 ± 3.1).

**Figure 1.**
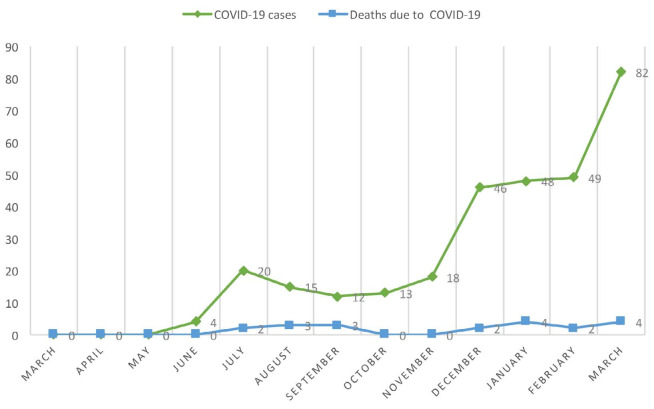
Monthly cases and deaths due to coronavirus disease 2019 (COVID-19): March 2020 to March 2021.

COVID-19 affected 339 patients (6.4%) during the study period. Out of these, 82 (24.2%) needed to be hospitalized and 23 died due to complications from the infection (case-fatality rate of 6.8%). The monthly numbers of diagnoses and deaths due to COVID-19 during the 12-month period are shown in Figure 1.

The demographic and clinical characteristics of the sample are described in Table 1. The patients hospitalized due to COVID-19 and those who died from COVID-19 were older and frailer, with more morbidities and more long-term use of medications. Furthermore, DM and cardiovascular diseases also were more prevalent in this group.

**Table 1.** Demographic and clinical characteristics of the sample

Demographic and clinical characteristics	Overall sample n = 5,295	With COVID-19 n = 339	Hospitalization due to COVID-19 n = 82	Death due to COVID-19 n = 23
**Age (mean ± SD)**	78.6 ± 9.4	77.7 ± 9.7	81.0 ± 8.1	82.6 ± 7.5
**Females (%)**	72.6	70.8	64.6	60.8
**Frailty (mean ± SD)**	16.8 ± 6.9	17.25 ± 7.01	19.6 ± 7.9	20.9 ± 8.0
**Multimorbidity (mean ± SD)**	5.4 ± 3.0	6.8 ± 3.2	7.3 ± 3.0	7.6 ± 2.6
**Medications with long-term use (mean ± SD)**	5.2 ± 3.11	5.5 ± 3.1	6.7 ± 3.0	6.6 ± 2.8
**Obesity (%)**	52.7	59.3	60.1	52.2
**Smoking (%)**	4.0	3.8	3.7	4.3
**Diabetes mellitus (%)**	27.5	32.2	47.6	52.2
**Respiratory diseases (%)**	7.9	9.7	8.5	4.3
**Cardiovascular diseases (%)**	9.0	10.0	15.8	17.4
**Chronic kidney disease (%)**	10.5	8.5	6.1	8.7
**Cerebrovascular disease (%)**	5.8	5.6	8.1	7.7

Frailty = values from Clinical-Functional Vulnerability Index - 20 (Índice de Vulnerabilidade Clínico Funcional – 20, IVCF-20); multimorbidity = number of diagnoses; SD = standard deviation; COVID-19 = coronavirus disease 2019.

Table 2 displays the results from the univariate and multivariate logistic regressions regarding associations between demographic and clinical characteristics and hospitalization due to COVID-19. Age, frailty, multimorbidity, long-term use of medications, DM and cardiovascular disease increased the odds of hospitalization due to COVID-19 in the univariate analysis (**Model 1**). Multimorbidity, number of medications with long-term use and DM also increased the odds of hospitalization due to COVID-19 regardless of sex, age and frailty (**Model 2**). An association of stronger magnitude was observed for DM (OR: 2.27; 95% CI: 1.45-3.56).

**Table 2.** Demographic and clinical characteristics associated with risk of hospitalization due to coronavirus disease 2019 (COVID-19)

Demographic and clinical characteristics	Hospitalization due to COVID-19	
	Model 1 OR (95% CI)	Model 2^a^ OR (95% CI)
**Age**	1.02 (1.00-1.05)	…
**Females**	1.46 (0.92-2.31)	…
**Frailty**	1.05 (1.02-1.09)	1.06 (1.02-1.09)
**Multimorbidity**	1.19 (1.12-1.27)	1.17 (1.09-1.26)
**Medications with long-term use**	1.16 (1.08-1.24)	1.13 (1.06-1.22)
**Obesity**	1.41 (0.90-2.20)	…
**Smoking**	0.90 (0.28-2.88)	…
**Diabetes mellitus**	2.42 (1.56-3.75)	2.27 (1.45-3.56)
**Respiratory disease**	1.08 (0.49-2.36)	…
**Cardiovascular disease**	1.92 (1.05-3.50)	1.60 (0.87-2.96)
**Chronic kidney disease**	0.54 (0.22-1.36)	….
**Cerebrovascular disease**	1.45 (0.66-3.18)	….

^a^ Adjusted for sex, age and frailty; except frailty, adjusted for sex and age; multimorbidity = number of diagnoses; frailty = values from Clinical-Functional Vulnerability Index - 20 (Índice de Vulnerabilidade Clínico Funcional – 20, IVCF-20); OR = odds ratio; CI = confidence interval.

Table 3 displays the results from the univariate and multivariate logistic regressions on associations between demographic and clinical characteristics and death due to COVID-19. The same characteristics associated with higher odds of hospitalization also increased the odds of death due to COVID-19, except for cardiovascular disease in the univariate analysis (**Model 1**). Multimorbidity (OR: 1.16; 95% CI: 1.03-1.32) and DM increased the odds of death due to COVID-19 (OR: 2.69; 95% CI: 1.79-6.14), even after adjusting for sex, age and frailty.

**Table 3.** Demographic and clinical characteristics associated with risk of death due to coronavirus disease 2019 (COVID-19)

Demographic and Clinical Characteristics	Deaths due to COVID-19	
	Model 1 OR (95% CI)	Model 2^a^ OR (95% CI)
**Age**	1.05 (1.00-1.10)	…
**Females**	1.71 (0.73-3.96)	…
**Frailty**	1.04 (1.02-1.14)	1.07 (1.00-1.14)
**Multimorbidity**	1.22 (1.08-1.37)	1.16 (1.03-1.32)
**Medications with long-term use**	1.16 (1.08-1.24)	1.09 (0.96-1.25)
**Obesity**	0.98 (0.43-2.22)	…
**Smoking**	1.08 (0.14-8.08)	…
**Diabetes mellitus**	2.88 (1.27-6.55)	2.69 (1.79-6.14)
**Respiratory disease**	0.52 (0.70 -3.90)	…
**Cardiovascular disease**	2.13 (0.72-6.28)	…
**Chronic kidney disease**	0.80 (0.18-3.46)	…
**Cerebrovascular disease**	1.36 (0.32-5.78)	…

^a^ Adjusted for sex, age and frailty; except frailty, adjusted for sex and age; multimorbidity = number of diagnoses; frailty = values from Clinical-Functional Vulnerability Index - 20 (Índice de Vulnerabilidade Clínico Funcional – 20, IVCF-20); OR = odds ratio; CI = confidence interval.

## DISCUSSION

In this retrospective cohort study among frail community-dwelling elderly people, older age and frailty increased the odds of hospitalization and death due to COVID-19. Our results also showed that, independently of the effects of age and frailty, multimorbidity and DM increased the odds of hospitalization and death. Furthermore, the number of medications with long-term use increased the odds of hospitalization due to COVID-19.

Advanced age has already been consolidated as a risk factor for death due to COVID-19,^18^ with a case-fatality rate of 19.3% among individuals over 65 years old,^19^ and a risk of death three times greater for those between 60 and 69 years old, compared with those between 50 and 59 years old.^20^ Frailty has also emerged as a factor associated with worse prognosis for older patients with COVID-19. From a cohort of 1,564 COVID-19 inpatients, with mean age of 74 years, it was shown that a more pronounced level of frailty increased the hospital stay and the risk of mortality, even after adjustments for age, sex, smoking and morbidities. The risk of death was four times higher among patients with the highest level of frailty on the Clinical Frailty Scale (hazard ratio, HR: 4.41; 95% CI: 2.90-6.71), compared with non-frail patients.^21^ From another cohort of 114 COVID-19 inpatients in Wuhan, China, with mean age of 67 years, it was shown that frail and pre-frail patients presented higher risk of developing severe disease (severe pneumonia or severe acute respiratory syndrome) within 60 days.^8^

Like frailty, the presence of morbidities has also been correlated with increased risk of death due to COVID-19. In a systematic review with a metanalysis on 58 cohort studies (122,191 participants), the risk of death among COVID-19 inpatients was doubled for patients with any morbidity, compared with those without any disease.^22^ In that study, DM also increased the risk of death 1.8-fold.^22^

Additionally, multimorbidity has been associated with higher mortality among COVID-19 inpatients. In a cross-sectional study with data from 1,591 patients hospitalized due to COVID-19 in Italy, it was demonstrated that patients who died from COVID-19 had higher multimorbidity, as measured using the Charlson Comorbidity Index.^23^ A retrospective cohort study with 5,621 COVID-19 inpatients in South Korea provided support for the idea that multimorbidity is a predictor of worse prognosis (admission to the intensive care unit, use of mechanical ventilation or use of extracorporeal membrane oxygenation) and mortality.^24^ A study in the United States using data from the Department of Veteran Affairs healthcare system, from 10,131 people infected with SARS-CoV-2, with a median age of 63.6 years, also showed that higher multimorbidity was associated with higher risk of death.^25^

While a growing number of studies has supported the notion that frailty and morbidity, including diabetes mellitus, are risk factors for adverse prognosis and death among patients hospitalized due to COVID-19, our study is one of the few to explore these risk factors among elderly people who were followed up on an outpatient basis. Therefore, our study provides an important contribution to investigation of prognostic factors relating to COVID-19. Moreover, it was possible to demonstrate that multimorbidity contributed to the COVID-19 prognosis regardless of frailty and age.

Nonetheless, the present study had some limitations relating to its observational design. Despite adjustments for potential confounding variables, the presence of residual confounders cannot be ruled out. Our use of a retrospective source of data might have compromised the quality of information, but we minimized this problem through the specificity of the electronic medical records used in our clinic. In the same way, even though the data used came from required fields in the electronic medical records, we cannot rule out the possibility that some information may have been lost due to inadequate and incomplete filling out of the electronic medical records.

## CONCLUSIONS

Our results help to identify independent risk factors for hospitalization and death due to COVID-19 among frail community-dwelling elderly people. This is of fundamental importance for planning healthcare actions in this population, which remains vulnerable to complications from SARS-CoV-2 infection.
